# Multiomics data integration, limitations, and prospects to reveal the metabolic activity of the coral holobiont

**DOI:** 10.1093/femsec/fiae058

**Published:** 2024-04-23

**Authors:** Amanda Williams

**Affiliations:** Microbial Biology Graduate Program, Rutgers University, 76 Lipman Drive, New Brunswick, NJ 08901, United States; Department of Biochemistry and Microbiology, Rutgers University, 76 Lipman Drive, New Brunswick, NJ 08901, United States

**Keywords:** bioinformatics, climate research, coral holobiont, data integration, microbiome, multiomics

## Abstract

Since their radiation in the Middle Triassic period ∼240 million years ago, stony corals have survived past climate fluctuations and five mass extinctions. Their long-term survival underscores the inherent resilience of corals, particularly when considering the nutrient-poor marine environments in which they have thrived. However, coral bleaching has emerged as a global threat to coral survival, requiring rapid advancements in coral research to understand holobiont stress responses and allow for interventions before extensive bleaching occurs. This review encompasses the potential, as well as the limits, of multiomics data applications when applied to the coral holobiont. Synopses for how different omics tools have been applied to date and their current restrictions are discussed, in addition to ways these restrictions may be overcome, such as recruiting new technology to studies, utilizing novel bioinformatics approaches, and generally integrating omics data. Lastly, this review presents considerations for the design of holobiont multiomics studies to support lab-to-field advancements of coral stress marker monitoring systems. Although much of the bleaching mechanism has eluded investigation to date, multiomic studies have already produced key findings regarding the holobiont’s stress response, and have the potential to advance the field further.

## Teaser

A review of the usage, limits, and potential of multiomics data applications when applied to the coral holobiont.

## Introduction

Whereas theories about how eukaryotic biological complexity arose are diverse, one common thread among them is the need for cooperation, interaction, and mutual dependence between multiple species (Bosch and McFall-Ngai 2011). In the case of corals, this involves billions of cells from every domain of life, commonly referred to as the holobiont. By virtue of the algal endosymbiosis and subsequent metabolic sharing between holobiont members, reefs have come to cover 255 000 km^2^ of the Earth’s surface (Spalding and Grenfell 1997) and accommodate an estimated 7000 marine species in oligotrophic waters (Fisher et al. 2015). Modern coral reefs protect coastlines from storms and erosion (Beck et al. 2018) while providing economic prosperity to local communities through food supply and tourism. The economic value of coral reefs is difficult to appraise, but even modest estimates ($350 000/ha per year) are 70 times higher than rain forests (Costanza et al. 2014).

**Figure 1.**
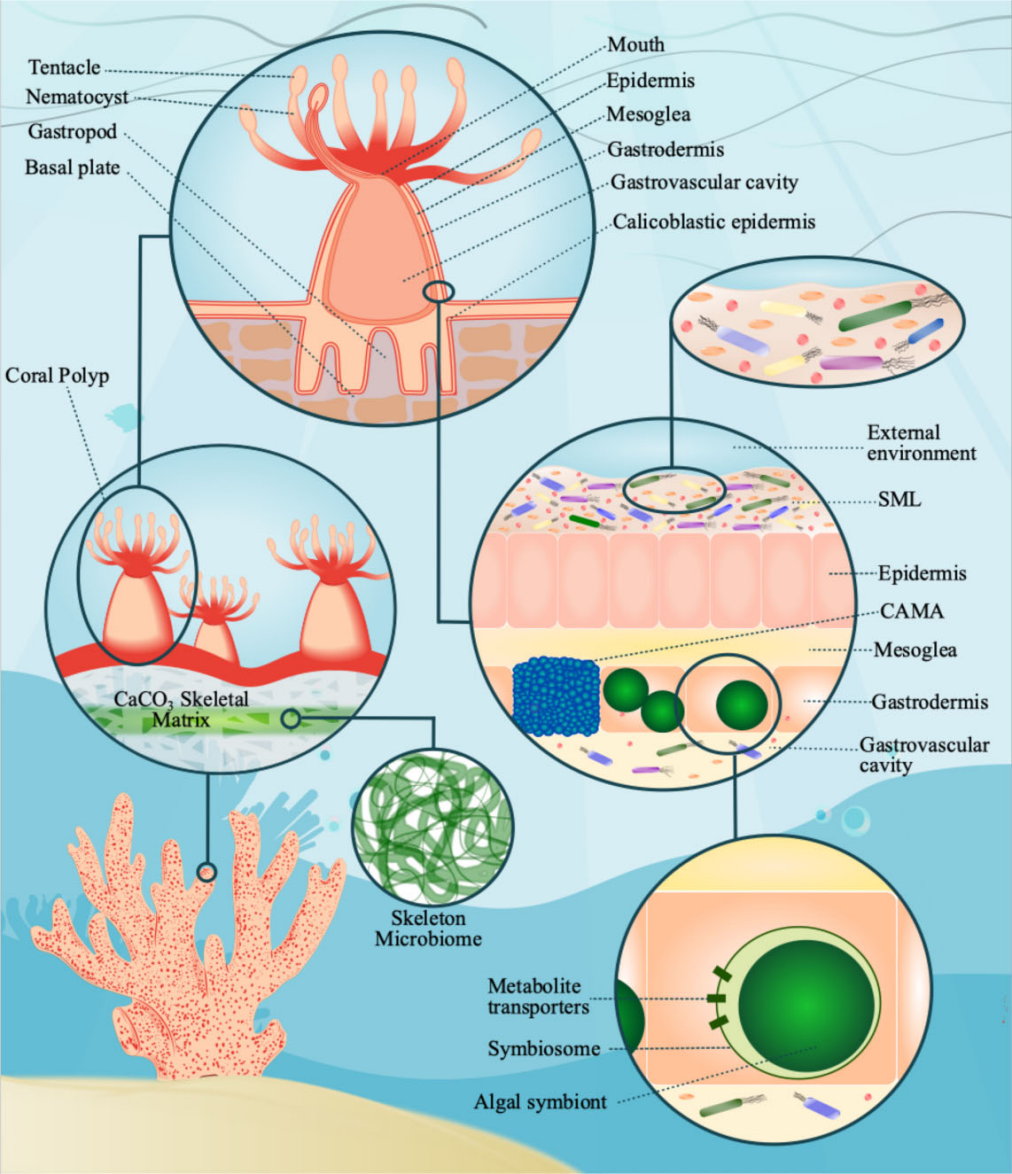
Illustration of a stony coral holobiont, displaying a cross-section of the coral polyp to show the detailed structure of the tissue layers, surface mucopolysaccharide layer (SML), and skeleton, in addition to the microbiome components associated with each feature of the polyp. Corals have radial body plans consisting of epidermal and gastrodermal epithelia, mesoglea, and a gastrovascular cavity. Stinging tentacles and nematocysts are used by the polyp to catch prey, mainly zooplankton. Coral animals, in conjunction with symbionts, secrete aragonite crystals from the base of the polyp to form the skeletal structure. Symbiodiniaceae inhabit the gastrodermis layer. Algal symbionts are encased in a host-derived membrane called the symbiosome, which allows the transport of metabolites between the alga and coral. Corals will acidify the symbiosome microenvironment (pH ∼4) in order to promote photosynthesis. The densest microbial community exists in the SML and includes many archaea, bacteria, and algal species, as well as viruses, as represented by an abundant array of microbial species. The SML is composed of glycoproteins and sulphated oligosaccharides connected via glycosidic linkages. It is enriched in nitrogen and organic material, providing a rich food source for the microbiome. In return, the SML microbial community protects the coral from invasive pathogens, cycles nutrients, and provides bioavailable forms of trace metals, vitamins, and other cofactors. The microbial community of the SML is distinct from microbial profiles of the adjacent seawater, indicating that the host exhibits control over its microbiome members. Because the SML regularly sloughs off from the coral surface, holobiont microbial communities must be dynamic and well-adapted to participating in this ecosystem. Endolithic prokaryotes, fungi, and filamentous algae associated with the skeleton are depicted as green filaments. The species in the genus *Ostreobium* are the most abundant members of the skeletal microbiome. Coral-associated microbial aggregates (CAMAs) reside in the epidermis and gastrodermis layers are represented as clumps of blue cells. Although CAMAs have been described as facultative symbionts, they are prevalent and abundant in coral tissue, suggesting they do help maintain coral fitness.

Corals have coevolved (Ritchie and Smith 2004, Rohwer and Kelly 2004) with diverse yet specific populations of microorganisms (Rohwer et al. 2002, Koren and Rosenberg 2006, Bourne et al. 2016). These species can either confer beneficial genes and traits (Lesser et al. 2007, Neave et al. 2017, Zhang et al. 2021) or cause disease and holobiont dysfunction (Garren et al. 2014, Ainsworth et al. 2017, Klinges et al. 2020). The coral microbiome is a combination of the microorganisms, varying in species and density, their combined genetic material, and their active metabolism (Wang et al. 2018). The core composition of this community is largely determined by the host (Kelly et al. 2014) to cultivate metabolic adaptations to local environmental conditions through the selection of beneficial genes (Ainsworth et al. 2017). The functions of the holobionts’ microbial component are known to provide protection against pathogens (Rohwer et al. 2002, Shnit-Orland and Kushmaro 2009), cycle carbon, nitrogen, sulfur, and phosphate (Bourne et al. 2016), and provide bioavailable forms of trace metals, vitamins, and other cofactors (Phillips 1984). To this end, the host possesses several distinct habitats suited for microbial residence (Fig. 1): the tissue (epidermis and gastrodermis; (Lesser et al. 2007), the gastric cavity (Herndl et al. 1985), the skeleton (Shashar et al. 1997), and the surface mucus layer (SML; Rohwer et al. 2002, Kooperman et al. 2007).

The loss of coral reefs is equivalent to the loss of rainforests in terrestrial systems. Although the holobiont has adapted and survived past climate fluctuations, current environmental changes caused by anthropogenic climate change, such as increased sea surface temperature and ocean acidification, have proven to be beyond anything since the last glaciation (Pandolfi et al. 2011). For the aforementioned reasons, there is a heightened focus on research dedicated to improving the resilience of coral reefs in the face of current and future stresses (Bourne et al. 2016), accelerating the need for a detailed understanding of holobiont group dynamics and responses to stress.

An integral part of the coral holobiont, especially during times of stress, are the algal endosymbionts. The role of the algal endosymbiont population in relation to bleaching susceptibility is multilayered, as bleaching risk depends on many aspects, such as the symbiont species present, (Glynn et al. 2001), the effect of endosymbiont density (Cunning and Baker 2013, Scheufen et al. 2017a), and the adaptation of the coral holobiont to the stress (Cunning and Baker 2013, Scheufen et al. 2017a,b, Barott et al. 2021, Vidal-Dupiol et al. 2022). The endosymbionts are dinoflagellates belonging to the family Symbiodiniaceae. Endosymbiont species differ in their growth rates, photosynthetic abilities, host specificity, number of chromosomes, and allozyme alleles (Blank and Trench 1985, Stat 2010, LaJeunesse et al. 2018). The symbiont community composition is distinct for each coral species at a given location (Howells et al. 2020, de Souza et al. 2022) and each profile confers varying traits to the coral animal (Wall et al. 2020, Torres et al. 2021). This is partially because symbiont species vary in their rate of proliferation (Stat 2010), control over host–algal nutrient exchange (Stat et al. 2008, Aranda et al. 2016), and photosynthetic rate during environmental stress (Davy et al. 2012, Scheufen et al. 2017a, Liu et al. 2018). It is important to note that endosymbiont physiology, particularly in terms of their photosynthetic rate, is itself mediated by the optical and biological interactions within the host, so defining the coral–symbiont–skeleton unit is essential when discussing photosynthetic rate (Scheufen et al. 2017a). Working with four different coral species, Scheufen et al. (2017a) found that combinations of skeleton morphology, tissue thickness, variation of coral pigmentation, species plasticity for changing symbiont content, dominant endosymbiont type, and symbiont cell pigmentation contribute to the rate of algal photosynthesis, and therefore the risk of bleaching. This study supported the idea that “pigment packaging,” or the distribution of pigmentation over cell area, within coral tissue is a coral–symbiont dynamic that presents a relevant species-specific component to various stress responses and bleaching risk. This change in vulnerability for corals provides a quantitative, mechanistic link between algal endosymbiont metabolism and the molecular basis for coral bleaching. However, the mechanism of coral bleaching remains unknown.

Beyond the algal endosymbionts, there is evidence that holobiont microbial communities are dynamic and well-adapted to participating in this ecosystem (Rohwer et al. 2001, 2002, Frias-Lopez et al. 2002, Reshef et al. 2006, Rosenberg et al. 2007, Bythell and Wild 2011). However, the advantages the microbiome affords the coral appear to fade when the holobiont is exposed to stress (Sunagawa et al. 2010, Littman et al. 2011). This destabilization is supported by a change in diversity (Thurber et al. 2009, Zaneveld et al. 2017) and a concomitant shift toward opportunistic microorganisms and pathogens (Ritchie 2006, Littman et al. 2011); yet, the controlling mechanisms behind the destabilization has yet to be unveiled.

**Figure 2.**
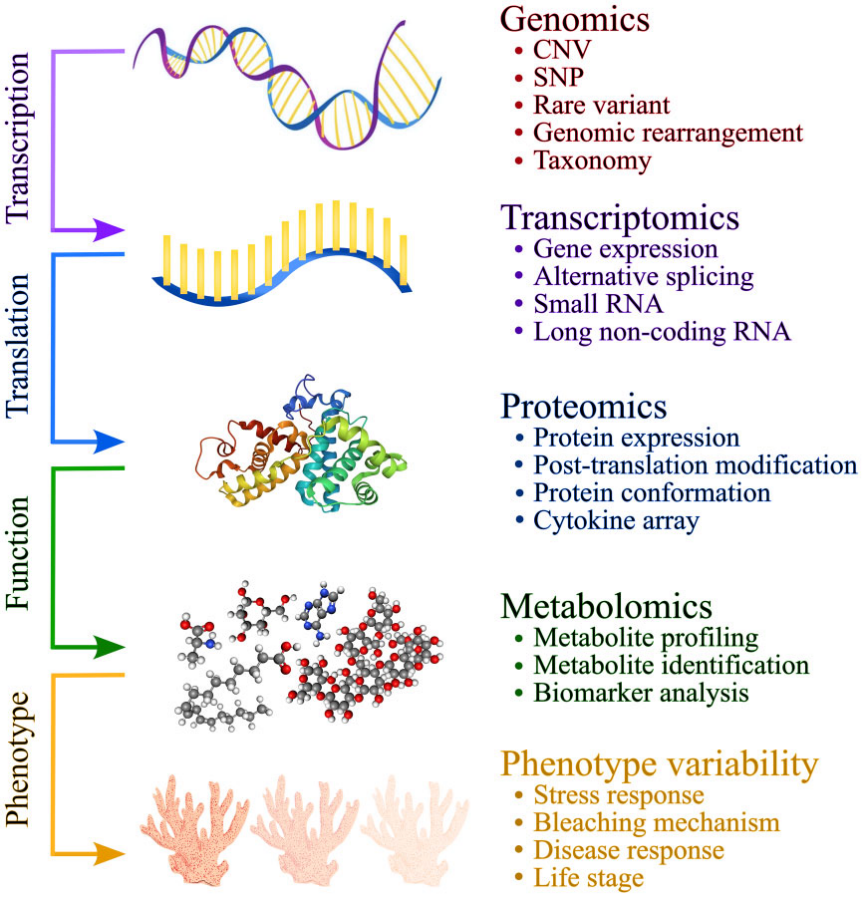
Schematic of the omics methods described in this review and information gained in each omics dataset. Genome, metagenome, and transcriptome data is produced via sequencing technologies. Genomics data (DNA) provides information about an organism’s potential, as well as genetic variation (copy number variants, single nucleotide polymorphisms, and genomic rearrangements), and in the case of metagenomics, species diversity and abundance. The transcriptome refers to the cell’s whole set of RNA transcribed from the genome at a given time to gain information about gene expression, as well as the regulation of gene expression. Proteomics and metabolomics utilize mass spectrometry for large-scale experimental analysis of the proteome and metabolome, respectively. Proteomics allows for expression profiling of proteins translated from RNA, in addition to acquiring information about their structures and regulation. Metabolomic data includes the most diverse set of compounds out of all the omics approaches as it is the final functional readout of the organism. Everything from the sample preparation to the instrumentation used will determine, which portion of the metabolome is measured for metabolite profiling. Both proteomic and metabolomic data can be analyzed for potential biomarkers of specific phenotypes.

Although the general interactions between the coral host and microbiome are documented, the controlling mechanisms and metabolic pathways operating within the holobiont stand unexplained at all stages of life. There is value in gaining a multifaceted understanding of the coral holobiont through multiomics approaches (i.e. genomics, transcriptomics, metabolomics, proteomics, and metagenomics; Fig. 2; Table 1) to answer questions about the roles of holobiont residents, how different stressors affect members of the holobiont, especially at different stages of coral development, the formation and deterioration of the symbiotic relationships, and ultimately identify biomarkers of coral stress and discover ways to encourage coral resilience. This review covers how these techniques have been applied to the coral holobiont, their limitations, and ways to overcome constraints, advance analyses, and ultimately optimize the data acquired.

**Table 1.** Details for the omics methods discussed in this review. Each omics method and the technologies used for each method are explained, and a description of the resulting data is provided.

Omics method	Omics description	Technologies or methods	Methods descriptions
Genomics and metagenomics	The study of all DNA within an organism (genomics), or within a group of organisms (metagenomics). This includes gene interactions with other genes and the environment. The data can be used to understand an organism’s identity, abundance, potential, and evolutionary history.	Marker gene analysis	Uses primers designed to bind to highly conserved genes or regions within these genes, amplification of the gene, and sequencing libraries for taxonomic profiling and quantifying of a species’ relative abundance.
		Shotgun sequencing	Involves randomly breaking up the DNA, which is then sequenced and reassembled using bioinformatic tools. This method can distinguish the genetic potential of microbiome members through providing genome-resolved phylogenic data.
		Culturomics	Relies on high-throughput culturing techniques, matrix-assisted laser desorption/ionization-time of flight (MALDI-TOF) mass spectrometry (MS), and 16S RNA sequencing to identify new species if initial identification fails.
Transcriptomics and metatranscriptomics	The study of all RNA within an organism (transcriptome), or group of organisms (metatranscriptomics), at a given time. The data provides information about gene expression, as well as the regulation of gene expression.	RNA-Seq	Uses next-generation sequencing to sequence RNA that has been isolated and reverse transcribed into cDNA, or complementary DNA. Library preparation can include steps to enrich RNA with 3′ polyadenylated tails (polyA selection) or filter RNA that binds to specific sequences, such as microRNA (miRNA). The resulting sequences are either assembled de novo or mapped to genomes. The data allow studies to gain information about changes in gene expression, alternative gene spliced transcripts, post-transcriptional modifications, gene fusion, and mutations/SNPs.
Proteomics and metaproteomics	The study of proteins within an organism or group of organisms (metaproteomics). Proteomics allows for expression profiling of proteins translated from RNA, in addition to acquiring information about their structures and regulation	Mass spectrometry	Proteins are first isolated from samples before proteins are prepared for measurement through MS. Two MS-based methods are currently used. The first uses 2D-electrophoresis to separate proteins within a sample, followed by selecting and staining proteins of interest, using based on differential expression or general abundance. The proteins are then identified using MS. The second method utilizes stable isotope tagging to label proteins differentially before the proteins are digested and the labeled peptides analyzed via tandem MS. Proteomics allows for expression profiling of proteins translated from RNA, the determination of what mRNA is translated into proteins, information about protein interactions, and the identification of any posttranslational modifications.
Metabolomics	The study of all metabolites within an organism or group of organisms. Metabolomic data includes the most diverse set of compounds out of all the omics approaches as it is the final functional readout of the organism.	Mass spectrometry	MS methods involve extracting metabolites from samples which are then analyzed by the MS instrument. Portions of the metabolome can be isolated to capture different kinds of molecules based on size or polarity. MS-based methods will obtain the most extensive metabolome coverage compared to NMR, with the most versatile technique incorporating High Performance Liquid Chromatography (HPLC). Liquid chromatography-mass spectrometry (LC-MS) will detect any polar, ionic, thermally labile, and nonvolatile organic compound that undergoes sufficient ionization. Specific MS signatures can be acquired through the addition of tandem MS (MS/MS), which improves metabolite identification. Gas–liquid partition chromatography (GLPC), commonly known as gas chromatography-mass spectrometry (GC-MS), is another analytical method able to measure an extensive range of analytes that are volatile and thermally labile.
		Nuclear magnetic resonance spectroscopy (NMR)	As with MS, metabolites are first extracted. NMR operates on the principle that nuclei are electrically charged and have spin, so when an external magnetic field (radio waves) is applied, the energy is transferred to the nuclei, promoting it to a higher energy level. Energy is emitted at the same frequency (as nuclear magnetic resonance) it was absorbed when the spin returns to its ground level and detected with radio receivers. Numerous NMR techniques are available, including onedimensional (OD) 1H NMR, two-dimensional (2D) NMR, and 13C NMR. NMR can detect all organic compounds with metabolite concentration proportional to signal intensities, albeit at lower sensitivities when compared to MS methods, especially for complex samples. However, NMR has the added advantage of elucidating extensive structural information to aid in unknown identification, chirality, and position specific isotope enrichment studies.

### Coral studies and omics techniques

There are many hurdles to effectively capture an accurate image of holobiont interactions and physiology. One major obstacle is the inability to separate holobiont members for individual, *in situ*, omics analyses. While metagenomics of the microbiome through its separation and/or enrichment is a worthwhile pursuit (Robbins et al. 2019), altered physiological states introduced during the time and processes required to separate and enrich microbial species call into question the accuracy of other omics techniques used to observe metabolic activity (i.e. transcriptomics, proteomics, metabolomics, and metadata). How this issue pertains to each omics method specifically is discussed in the ensuing respective sections.

### Microbial taxonomic identification techniques

To discern the identities of microbial holobiont residents, marker gene analysis (amplicon sequencing) and shotgun sequencing have historically been employed. Marker gene analysis relies upon primers designed to bind to highly conserved genes or regions within these genes, amplification of the gene, and sequencing libraries for taxonomic profiling and quantifying of a species’ relative abundance. For example, 16S rRNA (bacteria and archaea) and ITS2 (algal symbionts and fungi) region amplicon sequencing has been used to survey the composition of the coral microbiome and its flux with changing environmental conditions, seasons, and time (Sweet et al. 2011, Arif et al. 2014, Apprill et al. 2016, Wang et al. 2018, Dunphy et al. 2019, Maher et al. 2019, Klinges et al. 2022). However, this approach is not without error, as many biases are introduced during PCR amplification and sequencing. Namely, primers do not exhibit equal affinities for all DNA sequences because of GC bias or overall abundance of sequences present (i.e. the more a sequence is present, the more likely it is to be amplified, and therefore more likely sequenced; (Brooks et al. 2015). Moreover, these studies only allow speculation concerning the metabolism of the microbiome and its interactions with the coral host and surrounding environment.

Metagenomics can distinguish the genetic potential of microbiome members through providing genome-resolved phylogenic data (Thurber et al. 2009, Littman et al. 2011, Badhai et al. 2016, Cissell and McCoy 2021, Palladino et al. 2022). Again, this technique is not without its faults, particularly when applied to the holobiont. The microbiome-related sequences can be easily overwhelmed by the coral genome, which registers the majority of sequence data (Wegley et al. 2007, Robbins et al. 2019). For this reason, separation and enrichment methods may be required for metagenomics of the microbiome to be fruitful (Thurber et al. 2009, Robbins et al. 2019, Palladino et al. 2022).

Employing techniques developed in other fields is one way to propel coral microbial identification forward. One such example is culturomics, which was established by human gut microbiologists to identify unknown microbial species (Seng et al. 2009, Lagier et al. 2012, 2016, Diakite et al. 2019). Culturomics relies on high-throughput culturing techniques, matrix-assisted laser desorption/ionization-time of flight (MALDI*-*TOF) mass spectrometry (MS), and 16S RNA sequencing to identify new species if initial identification fails (Seng et al. 2009). Samples are first divided and diversified into various culture conditions for prolonged incubation times to stimulate the growth of species present at lower concentrations, or any specific taxa, before using MALDI-TOF MS to identify taxa (Lagier et al. 2018). The successful use of this time and cost-effective platform relies upon ever growing databases containing the protein mass spectra of each species, preferably from multiple isolates. When identification via MALDI-TOF MS is unsuccessful, 16S rRNA sequencing is then used to identify new species that are then added to the database. In one study, fecal samples were incubated with ethanol to enrich the abundance of spores, resulting in the growth of 137 sporulated bacterial species, of which 69 were new taxa (Browne et al. 2016). More recently, culturomics has been implemented beyond clinical microbiology to isolate 40 bacterial species from the planarian flatworm *Schmidtea mediterranea* (Kangale et al. 2021), 18 species from table salt, including one novel species (Diop et al. 2016), and 17 bacterial strains present in soil capable of degrading diesel oil, bitumen, and multiple polycyclic aromatic hydrocarbons (Chicca et al. 2022).

### Holobiont transcriptomics

The advent of sequencing transcriptomic responses has radically altered the analytical and experimental model of coral biology more than any other omics technique (Miller et al. 2011). Transcriptomic sequencing has granted a view of coral cellular processes during environmental changes (i.e. thermal, light, saline, and pH stress; (DeSalvo et al. 2010, Bay and Palumbi 2015, Davies et al. 2016, Aguilar et al. 2019, Savary et al. 2021, Ip et al. 2022), local stressors (i.e. pollution and sedimentation; (Yuan et al. 2017, Zhou et al. 2018, Poquita-Du et al. 2019, Xiang et al. 2019, Bollati et al. 2022) and disease (Libro et al. 2013, Vidal-Dupiol et al. 2014, Daniels et al. 2015, Frazier et al. 2017, Kelley et al. 2021, Traylor-Knowles et al. 2021). Poquita-Du et al. (2019) subjected *Pocillopora acuta* to both heat and sediment treatments. RNAseq data findings included the disruption of genes related to cilia assembly and disassembly, the downregulation of genes involved in cell adhesion, and highly unregulated innate and adaptive immune responses in samples exposed to heat and sediment stress compared to controls. When comparing *Orbicella faveolata* and *Montastraea cavernosa* colonies with and without stony coral tissue loss disease (SCTLD), which has infected almost one third of Caribbean coral species and half of Florida’s coral species to date (Skrivanek and Wusinich-Mendez 2020), Traylor-Knowles et al. (2021) discovered differentially expressed genes (DEGs) in the diseased corals were functionally enriched for pathways associated with hormone synthesis and signaling, such as Wnt and mTOR.

Studies utilizing transcriptomics have also examined basic coral biology to better understand coral reproduction (Williams et al. 2021b, Zakas and Harry 2022), life stages (Meyer et al. 2009, Portune et al. 2010, Siboni et al. 2014, Strader et al. 2018, Chille et al. 2021, Yoshioka et al. 2022), and biomineralization (Mass et al. 2016, Drake et al. 2021, Neder et al. 2022, Han et al. 2022). Strader et al. (2018) profiled *Acropora millepora* gene expression in embryos and larvae for 12 days postfertilization. There was a rise in larval response to settlement cues throughout development as well as the upregulation of signal transduction and sensory genes. In another study, while investigating how similar biomineralization mechanisms produce such varied morphological differences across four coral species—*Pocillopora damicornis, Pocillopora verrucose, Acropora muricata*, and *Montipora foliosa—*Han et al. (2022) found that carbonic anhydrase 2 was expressed at high levels universally. However, calcium ATPases and bicarbonate transporter expression levels fluctuated, which implies that Ca^2+^ and HCO_3_ transport rates contribute to morphological variances.

As far as transcriptomics has advanced coral research, issues related to conclusions based entirely on sequenced mRNA should not be disregarded. Assembling short reads without a reference genome sequence into a de novo transcriptome is complicated by paralogs, alleles, and other processes such as alternative splicing (Miller et al. 2011). Highly repetitive genomic regions (Miller et al. 2011) and polymorphism present in many corals (Shinzato et al. 2015, Drury et al. 2016, Quigley et al. 2020, Zayasu et al. 2021, Poliseno et al. 2022) specifically complicates the assembly process of corals. These impediments can be resolved with a high-quality genome, which exists for few coral species, but still does not establish the actual protein abundance present, as it has been shown that mRNA expression often does not equate to protein expression (Williams et al. 2023).

As the genes of the active microbial populace contribute to the genetic potential of the holobiont, the microbial metatranscriptome holds information regarding how the holobiont responds to external factors to identify ecologically relevant functions. Unfortunately, obtaining prokaryotic transcripts with adequate coverage of the coral microbiome is specifically challenging. This is because most transcripts originate from the coral animal, and as previously mentioned, microbial enrichment practices alter the metatranscriptome. The high cost of library preparation and sequencing compound this problem, as does the lack of standard methods for RNA extraction, storage, and preparation, which impact transcript and taxa recovery (Cleves et al. 2020). Further, even in simpler systems, there has been evidence that some microbial mRNAs are poorly translated because they have weak ribosomal binding sites (Liang et al. 2000). Therefore, beyond sequencing biases, the microbial metatranscriptome may not include mRNA involved in the holobiont phenotype and its response to perturbations.

Sequencing Symbiodiniaceae-specific transcriptomes is aided by the application of PolyA selection (Moitinho-Silva et al. 2014, Daniels et al. 2015), but most transcripts still belong to the coral host, and analysis of what transcripts remain is hindered by the lack of accurate reference algal genomes (Lin 2011). Symbiodiniaceae genomes are large (1–5 Gbp) with idiosyncratic features due to a combination of neutral selection and symbiotic associations varying by host specificity (Wisecaver and Hackett 2011, LaJeunesse et al. 2018, Forsman et al. 2020). Recent analysis of 15 Symbiodiniaceae genome sequences from species ranging from obligate symbiont to free-living revealed that >95% of core genes in *Symbiodinium* species and 83.56% of genes in *Breviolum, Cladocopium*, and *Fugacium* are “dark,” or do not share significant sequence similarity to any UniProt protein sequences (González-Pech et al. 2021). Therefore, even a significant number of conserved genes within coral symbionts are not annotated, further complicating analysis of metatranscriptomic data. These obstacles have been overcome by numerous studies (Bayer et al. 2012, Ladner et al. 2012, Gust et al. 2014, Li et al. 2020, Mohamed et al. 2020, Yoshioka et al. 2021, Yuyama et al. 2022) and can be aided by tools (PSyTrans) and published bioinformatic strategies (Meng et al. 2018). Mohamed et al. (2020) used a dual RNA-sequencing approach to characterize coral–symbiont (*Acropora tenuis* and *Cladocopium goreaui*, respectively) interactions during colonization by comparing transcript levels of cultured *C. goreaui* to that of *in hospite* cells. The analysis revealed that in a symbiotic state, immune-related and stress response genes were downregulated, but overall metabolism was upregulated.

### Holobiont proteomics

Although the presence of most proteins detected through proteomics will be captured as mRNA, metaproteomics data can answer questions about what mRNA is translated into proteins comprising the proteome, the direct quantity of proteins present, protein interactions, and any posttranslational modifications, which may be extreme enough to completely change the functionality of said protein (Vogel and Marcotte 2012, Liu et al. 2016). Proteomics has been applied to corals to study how the animal responds to environmental stress (Weston et al. 2015, Ricaurte et al. 2016, Stuhr et al. 2018, Mayfield et al. 2021, Lin et al. 2022), acclimates to unstable environmental conditions (Mayfield et al. 2018, Janech et al. 2021), counters disease (Gochfeld et al. 2015, Zhang et al. 2017, Ricci et al. 2019), and forms their aragonite skeletal structures (Conci et al. 2020, Drake et al. 2020, Zaquin et al. 2021, 2022).

One study utilizing nine coral species from Key West, Florida reefs, all highly susceptible to SCTLD but varying in their disease dynamic, found that the downregulation of green fluorescent proteins in infected corals was consistent regardless of the coral species, representing its potential as a marker of SCTLD progression. (Janech et al. 2021). Conci et al. (2020) applied proteomics to compare the biomineralization process of calcitic octocoral species (*Tubipora musica* and *Sinularia* cf. *cruciata*), an aragonitic octocoral species (*Heliopora coerulea*), and an aragonitic scleractinian coral (*Montipora digitata*). The analysis presented little commonality when comparing the polymorphs, except for the presence of carbonic anhydrase CruCA4 in both calcitic and aragonitic species, and a galaxin-related protein and hephaestin-like protein present in both octocorals and scleractinians. Phylogenetic analysis indicated that out of the three proteins, only the hephaestin-like protein shared a common origin for these coral species.

Metaproteomics has the ability to study the holobiont as a whole (Maron et al. 2007), while also permitting the identification of a protein’s source without the need for host and microbial separation. The spectra, once processed, can be matched against databases, high quality transcriptomes, or genomes to link the expressed protein to a particular species, and therefore analyze host–microbial interactions. Microbial proteins and their products affect host physiology and other microbes present in the microbiome (Schweppe et al. 2015, Rolig et al. 2018), so the application of metaproteomics to identify and quantify microbial proteins permits a more accurate depiction of the metabolic functions of the holobiont. Large-scale studies to identify and quantify proteins expressed by the coral holobiont through high-resolution MS is a fairly recent method employed by coral researchers, thus far mainly to the coral host and algal symbionts (Petrou et al. 2021, Pei et al. 2022, Sun et al. 2022).

Petrou et al. (2021) utilized tandem MS to determine that proteins related to photosynthesis and energy production were downregulated without signs of oxidative stress, despite lipids stores in the symbiont increasing 2-fold, indicating the symbiont responded to heat stress by reducing carbon translocation to the coral animal. In another study, tandem mass tag labeling and nano LC-MS/MS analysis was used to determine larval and symbiont response to thermal stress and *p*CO_2_ (Sun et al. 2022). Again, many photosynthesis-related proteins were downregulated in the symbionts under thermal stress [photosystem (PS) I reaction center subunits IV and XI, oxygen-evolving enhancer], in addition to proteins present in the Calvin cycle (phosphoribulokinase) and the C_4_ pathway (phosphoenolpyruvate carboxylase), suggesting symbionts reduce carbon fixation when exposed to elevated temperatures. In contrast, under high *p*CO_2_ conditions, the expression of PS I iron–sulfur center proteins were upregulated. These findings were corroborated by Krämer et al. (2022).

There are instances of applying proteomics to individually cultured members of the microbiome (Chan et al. 2022, Pogoreutz et al. 2022). Through proteomics, it was shown that *Endozoicomonas montiporae*, a coral bacterial group that rapidly decreases in abundance during thermal stress events, positively expressed heat shock proteins and negatively expressed many antioxidant defense proteins (Chan et al. 2022). *Endozoicomonas montiporae* cultures were also incubated with coral lysates and exposed to heat stress. Some proteins were only differentially expressed with the combination of thermal stress and host presence, signifying *E. montiporae* protein expression is affected by heat-induced host factors. In a second species of *Endozoicomonas, E. marisrubri*, proteomic response to tissue extracts of its native host, *Acropora humilis*, was assessed. Pogoreutz et al. (2022) found that vitamin B1 and B6 biosynthesis, as well as glycolytic processes, were upregulated in response to holobiont metabolism, suggesting that symbiotic lifestyles of *Endozoicomonas* involve a modulation of host immunity.

Several platforms and tools exist to process metaproteomic data for all biological systems beyond model organisms, such as Galaxy-P (Jagtap et al. 2015), MetaProteomeAnalyzer (Muth et al. 2018), MetaQuantome (Easterly et al. 2019), and PromMetaLab (Cheng et al. 2017). Nevertheless, proteomics in general does not provide information about expressed noncoding elements or what genes are involved in the production or regulation of proteins (Graves and Haystead 2002), and only a subset of the total proteome can be measured at a time (Beck et al. 2011). Metaproteomic studies involve additional challenges. To distinguish between host and microbial proteins, gene sequences specific to the host and microbiome are needed, yet host and undigested food proteins can contaminate the microbial proteome (Isaac et al. 2019). Additionally, there is not a coral microbiome protein database, so many detected microbial proteins will not be identified with the use of general databases (Charubin et al. 2020). Even with highly populated microbial protein databases, an estimated 32%–40% of microbial proteins are annotated as “unknown function” (Goodacre et al. 2014, Poudel et al. 2021) expanding complications with regards to the analysis of microbial metaproteomic data.

### Holobiont metabolomics and identifying metabolite origins

Metabolomics is a rapidly emerging field providing a direct, functional readout of an organism’s physiological state (Geier et al. 2020) through a combination of strategies to identify and quantify cellular metabolites. Metabolomics employs sophisticated analytical instruments with statistical and multivariant methods to extract data for interpretation (Roessner and Bowne 2009). There exists a strong correlation between metabolites and the phenotype (Fiehn 2002), enabling meaningful studies of responses to biotic and abiotic stress.

Metabolomics has helped determine chemical signatures of biochemical responses in the holobiont (Raina et al. 2013, Hillyer et al. 2017, 2018, Lohr et al. 2018, 2019, Farag et al. 2018, Ochsenkühn et al. 2018, Williams et al. 2021a). Through polar metabolic profile analysis of *Montipora capitata* and *P. acuta* collected through high performance liquid chromatography-mass spectrometry (HPLC-MS), a collection of dipeptides (arginine–glutamine, alanine–arginine, valine–arginine, and lysine–glutamine) produced during thermal stress was discovered (Williams et al. 2021a). The data recovered was also able to expand on previously known coral derived metabolites, montiporic acids (MAs), finding that MAs were present in coral tissue, not just coral eggs, and produced by species outside of the *Montipora* genus (Hagedorn et al. 2015). Another method, gas–liquid partition chromatography (GLPC), coupled with a ^13^C isotope tracer was utilized to track autotrophic carbon during exposure to thermal stress intended to result in bleaching (Hillyer et al. 2018). Fluctuations of carbohydrate and fatty acid metabolism, lipogenesis, and homeostatic responses to thermal, oxidative, and osmotic stress were observed. Though it was widely accepted that algal symbionts were responsible for dimethylsuphoniopropionate (DMSP) production in the holobiont (Stefels 2000), which subsequently serves as a sulfur and carbon source for marine bacteria (Todd et al. 2007), DMSP was found in asymbiotic coral juveniles through the application of quantitative nuclear magnetic resonance (qNMR) analysis (Raina et al. 2013). DMSP levels in newly settled asymbiotic coral juveniles increased up to 54% over time, and once juveniles were exposed to thermal stress, this level increased up to 76%.

Metabolomic studies have the power to illuminate the complex, multifarious biological processes, which define the coral holobiont’s essential, responsive, and adaptive functions, but any subset of metabolomics is intrinsically challenging due to the complexity at the heart metabolomic studies. Sample preparation, the reactivity of primary and secondary metabolites, structural diversity, peak misidentification, and “dark” metabolites dictate what metabolites are detected and usable for analysis (Lu et al. 2017). This is further complicated by the holobiont structure, which rests upon metabolic exchange amongst holobiont members and is itself a response to symbiosis (Stat et al. 2008). Separating holobiont members for individual metabolomic studies still cannot resolve, which member of the holobiont is responsible for the production of a given metabolite. However, this can be answered through applying approaches, which utilize labeling, such as stable isotope probing, pulse chase experiments, isotope arrays, and bioorthogonal noncanonical amino acid tagging. These techniques may detect biochemical transformations of the microbiome, such as identifying active microbial participants and ascertaining the relative contributions the host and microbiome have on carbon, nitrogen, or sulfur cycling (Engelberts et al. 2021), but they cannot determine the directionality of metabolite allocation.

Resolving substrate directionality may be possible through the advancements in microscopy techniques, such correlative light and electron microscopy and light sheet fluorescence microscopy (LSFM), which combine 3D *in situ* microscopic imaging with chemical and antibody probes to profile and visualize cells under physiological conditions, such as cell proliferation, hypoxia, or apoptosis (Geier et al. 2020). For example, LSFM produces images of thin optical sections at high speed and is suitable for observing the inner architecture of the holobiont without physical sectioning to examine species composition, relative abundances, and physiologies of microbiome members through *in situ* hybridization of DNA probes coupled with immunofluorescence of antibodies specific to key proteins or enzymes responsible for target metabolite production (Parthasarathy 2018). These approaches would also boast the opportunity of achieving a mechanistic understanding of the fundamental processes by which the coral host forms and maintains symbiotic associations with inter- and intracellular microbial species, in addition to how these metabolic responses alter during stress and dysbiosis. As LSFM would be hindered by the opaqueness of the skeleton, this approach may be best suited with the coral model *Aiptasia*, or with coral larvae.

## Network-based omics data integration

Network-based methods have been commonly applied in the coral field and hold great promise for analyzing large datasets by shrinking the dimensionality of the data. Network biology uses tools derived from graph theory to construct useful data structures between pairs of components in a system to investigate the interactions or relationships between said components (Koutrouli et al. 2020). These associations are illustrated as edges connecting the components, with components represented as nodes, and graph measures such as betweenness, degree, and centrality used to interpret meaningful biological relationships (Emmert-Streib and Dehmer 2011).

From a methodological perspective, networks are defined by their incorporation of Bayesian methods. Bayesian networks are composed of the graph and a local probability model incorporating *a priori* data (e.g. data probability distribution, and parametric or nonparametric) into the modeling scheme (Heckerman 1998). NonBayesian (NB) networks are either constructed by correlation analysis (Menichetti et al. 2014) or utilize interactions defined by molecular data (Aerts et al. 2006). From a biological perspective, networks may utilize known interactions (PPI networks; Bersanelli et al. 2016), but this is not necessary for all network types (coregulation and coexpression). Networks, which do not rely on known interactions specific to a given species are especially useful in corals because of the current lack of supporting data available to nonmodel species (Williams et al. 2023). These include gene and protein coexpression networks, metabolite correlation networks, and microbial-specific networks. The following sections will discuss how the different network types have been applied to coral research to understand the coral holobiont interactions, as well as characterize new metabolites and identify areas lacking within coral research.

### Gene and protein coexpression networks

Gene coexpression networks have become a common approach in the coral field (Wright et al. 2015, Reyes-Bermudez et al. 2016, Rose et al. 2016, Ruiz-Jones and Palumbi 2017, Thomas et al. 2019, Alves Monteiro et al. 2020, Dixon et al. 2020, MacKnight et al. 2022) due to the emergence of the WGCNA (weighted gene coexpression network analysis) R package (Langfelder and Horvath 2008). WGCNA constructs NB coexpression networks through pairwise correlations and eigengenes, where nodes correspond to gene expression profiles and edges represent gene expression pairwise correlations. Module assignment is achieved through hierarchical clustering of the expression data. The eigengene of each module can be correlated to the sample traits (e.g. genotype, experimental condition, and location) to identify modules of interest. Eigengenes themselves can be considered a representative of their given module’s gene expression profile, greatly reducing the size and complexity of transcriptomic data (Langfelder and Horvath 2008).

With *Acropora digitifera*, Reyes-Bermudez et al. (2016) performed quantitative RNA-seq analysis on embryonic, larval, and adult samples. WGCNA analysis identified three coexpression modules of interest; during embryonic and larval transitions, modules corresponded to cellular fate and morphogenesis, whereas the transition of larval bodies to adult stages is defined by a switch in lifestyle and regulating polyp processes. MacKnight et al. (2022) found modules correlated to lesion progression rates of seven Caribbean coral species infected with white plague disease were dominated by immune responses and cytoskeletal arrangement. When applied to gene expression profiles of *Acropora hyacinthus* colonies collected following a natural bleaching event, some module eigengenes reflected expression patterns impacted 12 months after the event (Thomas and Palumbi 2017).

By slightly augmenting WGCNA, coexpression networks can be built for proteomic data (Zhang et al. 2016, 2018, Pei et al. 2017, Nishimura et al. 2021). The same basic concepts of WGCNA apply to proteomics; protein or peptide expression profiles represent nodes and edges correspond to pairwise correlations. A comprehensive understanding of WGCNA and the proteomic data itself is recommended to correctly implement the methods to construct a proteomic coexpression network. For instance, the “goodSamplesGenes” function should be applied to remove proteins with >50% of missing entries (Wu et al. 2020) and signed networks, where positively correlated proteins that correspond to modules, rather than unsigned networks, are suggested (Langfelder and Horvath 2008). Moreover, protein-based and peptide-based proteomic datasets have to be treated differently, as peptide-based datasets may result in concordant protein modules (Gibbs et al. 2013), but protein-based datasets may have related proteins cluster separately due to different overall regulation trends (Pei et al. 2017).

### Metabolomic networks

Experimental network strategies for metabolomic data include association networks (NB) and molecular similarity networks (Bayesian; Amara et al. 2022). Metabolite correlation networks use quantitative information, such as normalized ion counts, to represent interactions (edges) between metabolites (nodes). In these networks, nodes are metabolic intermediates in biological pathways, possibly steps in biological synthesis or demonstrating a dependence between multiple processes (Morgenthal et al. 2006). Generally, correlation networks serve as a wonderful complement to measuring discrete metabolite levels because they analyze the complete dataset and provide links between metabolic pathways (Perez De Souza et al. 2020). The major advantages of applying this method to the coral holobiont is that the previous mapping of biological pathways and metabolic relationships are not required. As already discussed, identifying metabolic origins, as well as specific microbial holobiont members, at a given time is a difficult feat. This approach could generate holobiont-specific metabolic interactions to be validated by future studies without much preceding information, although the addition of other omics data can help strengthen hypothesis generation (Williams et al. 2021a).

Correlation networks applied to untargeted metabolomic data are most useful when a significant number of metabolites in the network are chemically defined. Unfortunately for the coral holobiont, the majority of its compounds have a level four identification, or are unknown and do not match to databases through conventional techniques (Salek et al. 2013). Molecular similarity networks, therefore have a place in coral research when MS is used because they aid in the identification of unknown compounds by assuming similar fragmentation patterns result from related structures (Ramos et al. 2019). For this reason, they can be thought of as spectra similarity networks, where spectral pairs are compared by dot product calculations after vectorization in *n*-dimensions (Yang et al. 2013). The cosine angle connecting the vectors then dictates the degree of spectral similarity. A value of one represents an identical match, but any value above 0.7 will set significant edges and produce modules that contain metabolites capable of attaining identification through their association with similar, known metabolites (Perez De Souza et al. 2020).

The Global Natural Products Social Molecular Networking database, first curated by Wang et al. (2016), provides a platform for open-source mass spectral data sharing that has been incorporated into processing tools capable of producing molecular networks, such as mzMine2 (Pluskal et al. 2010), OpenMS (Röst et al. 2016), MeMSChem (Hartmann et al. 2017), and feature-based molecular networking (FBMN; Nothias et al. 2020). FBMN has been incorporated into coral studies to show an enrichment of tyrosine derivatives, oleoyl-taurines, and acyl carnitines in the coral exometabolome compared to the algal exometabolome, demonstrating a mechanism for reef nitrogen and phosphorous-recycling (Wegley Kelly et al. 2022) and the accumulation of betaine lipids with varying degrees of saturation produced by symbionts in corals that have never bleached compared to apparently healthy corals that had bleached 4 years prior, possibly due to betaine lipids with a higher degrees of saturation providing increased thermal stability to algal membranes (Roach et al. 2021).

### Protein–protein interaction networks

The STRING (Search Tool for the Retrieval of Interacting Genes; Heidelberg, Germany) database (Szklarczyk et al. 2022) is commonly used to generate protein–protein interaction (PPI) networks (Bayesian). STRING does support the coral species *Stylophora pistillata*, but these interactions do not have supporting data at this time and, to date, not many coral analyses have relied on PPI networks produced via STRING (Maor‐Landaw et al. 2014, Kaniewska et al. 2015, Ishibashi et al. 2021). Wong et al. (2021) generated a PPI network with proteomic data from *Platygyra carnosa* affected by coral skeletal growth anomaly, but annotated proteins were mapped using the human PPI database, rather than a coral-specific database.

### Coral research network analysis

One additional study worth mentioning constructed undirected weighted networks to visualize 50 years of research related to coral disease (Montilla et al. 2019). For this, the disease studied, host genus, marine ecoregion, and research objectives were set as nodes, or vertices, and their weighted co-occurrence (i.e. co-occurrence frequency) within a paper visualized as edges through the R package *igraph* (Csardi and Nepusz 2006). The network showed that almost half of research efforts focused only on five diseases affecting five species in five locations. Black Band Syndrome (BBS) was by far the most studied disease, but mainly in Florida and parts of the Caribbean. Ultimately, the data demonstrated that a limited range of approaches, such as immune responses to *Aspergillosis* and 16S sequencing for BBS, rather than more comprehensive studies, have thus far been considered to understand each coral disease.

### Microbial-specific networks

In microbiome studies, nodes can be biological (i.e. taxa, genes, and metabolites), environmental (i.e. pH, temperature, and radiation), or host (i.e. antioxidant capacity, respiration, and calcification) features (Jiang et al. 2019). When applied to microbiome multiomics data, network methods can establish how information flows amongst community members and the entire coral holobiont (Röttjers and Faust 2018). For instance, networks can be constructed and visualized to illuminate microbe–microbe or microbe–host interactions (Faust and Raes 2012), associate microbial members with metabolite production or cycling (Bouslimani et al. 2015), and ultimately lend perspective as to how environmental changes affect the microbial population (Kint et al. 2010, Alivisatos et al. 2015, Blaser et al. 2016, Maier et al. 2017).

Though there are few incidents of constructing networks to visualize aspects of the coral microbiome, those studies have provided insight into the dynamics of members who have evaded culturing techniques (Soffer et al. 2015, Lima et al. 2020, Maruyama et al. 2021). Soffer et al. (2015) utilized a network of phage–bacteria interactions (microbial 16SrRNA amplicons and phage metagenomes) found to be associated with *Orbicella annularis* colonies from the US Virgin Islands experiencing an outbreak of white plague during a bleaching event. Six phage strains exclusively interacted with two bacterial species enriched in the diseased corals (*Rhodobacterales* and *Campylobacterale*), suggesting such phages could provide phage therapy during white plague outbreaks to control additional opportunistic bacterial infections.

In another instance, shotgun metagenomic data collected from inner and outer reef colonies of *Pseudodiploria strigosa* in Bermuda was used to construct microbial networks of the surface mucopolysaccharide layer (SML) taxa (Lima et al. 2020). The networks for the SML microbiome at each reef zone were distinct from each other, with the outer reef network being more tightly connected compared to inner reef networks, indicating that the outer reef microbiomes have an increased community network structure. However, the SML networks for the inner reefs demonstrated a higher betweenness centrality, or contained several microbial taxa (*Deinococci, Methanomicrobia*, and *Alphaproteobacteria*) significantly enabling the connectivity of the community network. These networks, along with simulations of the annual temperature fluctuations at each reef zone, were used to build a model of the SML microbiome. The model ultimately showed that microbial network profiles locally and generally highly impacted the SML microbiome, although not as strongly as temperature.

## Non-network multiomics data integration approaches

Every level of omics data is a proxy for holobiont functions, but their individual analysis is limited to correlations, rather than reflecting causative processes. When omics data from more than one technique are produced for the same sample set, integration can provide useful insights into the flow of biological information within the system (Tarazona et al. 2018), and thus can help unravel divisions between genotype and phenotype (Subramanian et al. 2020). There are numerous multiomics data integration reviews covering different integration techniques (Beale et al. 2016, Bersanelli et al. 2016, Hasin et al. 2017, Huang et al. 2017, Pierre-Jean et al. 2020, Picard et al. 2021, Kang et al. 2022, Maghsoudi et al. 2022) for each data type (Berger et al. 2013, Kristensen et al. 2014, Pinu et al. 2019, Graw et al. 2021, Jendoubi 2021, Kim and Kim 2021) and system (Tagu et al. 2014, Wang et al. 2019, Jamil et al. 2020, Son et al. 2020, Daliri et al. 2021, Layton and Bradbury 2022) from which the coral field can draw knowledge. Examples of host–microbial data integration from the coral field, along with three tools designed for nonmodel organism data integration are outlined below.

### Host–microbial data integration

The combination of microbial identification and host metabolic activity can uncover how the host’s phenotype is influenced by microbial species (Zhang et al. 2019). Microbial and animal integral synergies are not limited to corals, allowing coral research to prosper by looking to other systems for guidance. Human–microbiome studies have generated three integrative approaches: metagenomics to metatranscriptomics, metaproteomics, and metabolomics (Zhang et al. 2019). When studying indigenous microbial communities of the holobiont, integrating microbial metagenomics with host omics data can advance biological interpretation of biological pathways, organic fluxes, and symbiotic relationships associated with specific microbial partners (DeSalvo et al. 2010, Daliri et al. 2021, Guo et al. 2021, Santoro et al. 2021, Savary et al. 2021, Voolstra et al. 2021, Lin et al. 2022, Rädecker et al. 2022).

In a landmark study, DeSalvo et al. (2010) showed that transcriptomic states of the coral *Montastraea faveolata* collected under thermal stress clustered more strongly based on symbiont genotype and physiological parameters rather than experimental condition. More recently, Voolstra et al. (2021) compared gene expression (coral and algae) and bacterial community composition of robust corals from the northern and central Red Sea (NRS and CRS, respectively). The coral and algal transcriptomic responses of NRS colonies were strong and the bacterial community assemblages changed considerably. This was supported by separate findings that gene expression and microbiome composition of *S. pistillata* collected from the NRS rapidly responded to thermal stress, but >94% and >71% of coral and algal genes, respectively, returned to their baseline levels during recovery, as long as thermal stress did not exceed 32°C (Savary et al. 2021). At temperatures above 34°C, coral mortality reigned and microbial diversity dwindled, becoming dominated by opportunistic species, demonstrating a lethal threshold for the most stress resistant species in the NRS.

### Tools for host-microbial data integration advancement

Current bioinformatic workflows do not commonly support the integration of multiomics data levels when analyzing the microbiome, especially for nonmodel organisms. gNOMO is an example of a new, complete bioinformatic pipeline designed for nonmodel organisms, capable of integrating up to three omics levels (metagenomics, metatranscriptomics, and metaproteomics) for the microbiome and host in parallel (Muñoz-Benavent et al. 2020). This type of tool could not only advance microbial population analysis, but allow for physiological investigations within the microbiome’s biological context. gNOMO was applied to experimental data collected from the complex microbiome affiliated with the cockroach *Blattella germanica*, which is comparable to the coral animal in that *B. germanica* has an endosymbiont in addition to an expansive gut microbiome, to determine the source (i.e. host or microbiome) of key enzymes present in the nitrogen metabolic pathway (Lopez-Sanchez et al. 2009, Carrasco et al. 2014). The results after integrating all three data levels proved that while the host does produce four key enzymes present in these pathways, the microbiome possessed and expressed the majority of enzymes present in nitrogen metabolism (Muñoz-Benavent et al. 2020).

Biochemical reactions are the connection between genes and metabolites. This basic, fundamental fact of life can be exploited to advance knowledge of coral physiology and stress response. Metabolite Annotation and Gene Integration (MAGI) software integrates metabolomics and gene data sets into a biochemical reaction network. The software provides metabolite identification, gene annotation, and generates metabolite—gene associations using various reaction databases (Erbilgin et al. 2019). MAGI was recently applied to data from a thermal stress experiment (Williams et al. 2021b). From the metabolites and genes differentially expressed during thermal stress compared to ambient conditions, a suite of redox reactions and antioxidant responses were found linked to thermal stress, possibly demonstrating how the coral animal attempts to limit oxidative stress. The analysis also provided evidence of sex steroid dysregulation, which was especially useful to explain the disrupted spawning event that occurred during the experiment.

IntegrOmics is a tool to integrate two types of omics data to visualize sample similarities and find correlations between datasets (Lê Cao et al. 2009). This tool relies on correlations between molecular components followed by sPLS (sparse partial least squares) regression. One important requirement for this tool is that each omics dataset be generated from the same samples. Compared to other integration tools, integrOmics is implemented through R and relatively simple with its methodology. Additionally, there are no limits on the omics data types analyzed, making it a favorable tool for coral multiomics integration. Many examples of its usage exist in the literature (Bassi et al. 2018, Li et al. 2022), especially for host–microbiome studies (Stewart et al. 2017, Sovran et al. 2019, Xia et al. 2022).

## Considerations for design of holobiont multiomics studies

Multiomics data analysis and integration are not possible without a comprehensive experimental design from the onset. The design of a multiomics experiment includes identifying the questions a study is capable of answering, what types of data are needed, and an integration strategy to analyze, interpret, and visualize the data (Tarazona et al. 2018, Santiago-Rodriguez and Hollister 2021). Perspective and opinion papers discussing best practices and ideas to unify coral studies are available (McLachlan et al. 2020, 2021, Grottoli et al. 2021, Pratte and Kellogg 2021, Gómez-Campo et al. 2022, Nielsen et al. 2022, Thurber et al. 2022, van Woesik et al. 2022), and their suggestions related to multiomics studies are summarized below, with the addition of recommendations not present in coral literature.

### Requirements for holobiont multiomic experiments

The first requirement for any omics integration study is that all omics and meta data be collected from the same samples (Cavill et al. 2016). Large enough samples should be chosen to procure sufficient amounts of each extract and whatever meta data is needed. Fragments, >9 cm^2^, have correspondingly been shown to improve accuracy of bleaching experiments (Nielsen et al. 2022). Although there is evidence that sampling mucus has a benign effect upon the coral (Zaneveld et al. 2016), it is not advisable to resample tissue from the same nubbin during time point analysis. This is because clipping the nubbin will instigate an immune wound healing response that will obfuscate data analysis (Palmer and Traylor-Knowles 2012). Preserving the entire nubbin at once with limited contact is the soundest way to limit additional stress placed upon the nubbin and sample–sample variance.

Concerning tank experiments, as with nonmultiomics studies, an acclimatization period is crucial for experimental and control corals. The initial clipping and handling will elicit a stress response. Further, corals are adapted to their given environment. This includes variables such as temperature, light, and pH. Even within a single colony, nubbins will be adapted to various light levels (Gómez-Campo et al. 2022). Nubbins need to be acclimatized to initial tank conditions (light, pH, and temperature), and again to the experimental conditions. Samples should be monitored for tank and experimental acclimatization. How monitoring is performed will vary depending on the study’s limits and questions it is attempting to ask. Again, it is important to remember that clipping nubbins will cause a stress response, so either choosing techniques that would not utilize coral tissue, and thereby inflict stress on the animal, or collecting additional samples for measurements should be determined when planning the experiment. Regardless, it is vital to ensure samples are acclimated, using measurements such as maximum photochemical efficiency (Fv/Fm; Gómez-Campo et al. 2022).

Recent studies have demonstrated the significant level of divergence between genotypes of the same species (Stephens et al. 2021). For this reason, it is recommended that genotypes not be mixed within time points or conditions. Enough nubbins from a single genotype should be available for enough replicates to be collected from each timepoint under every given condition. However, repeating the experiment with multiple genotypes will help to unravel how genotype affects the data (Lohr et al. 2019, Williams et al. 2021a, 2023).

Omics data cannot be accurately analyzed without proper metadata to define the biological states of each sample. Some covariate data such as symbiont genotype identification via qPCR (Silverstein et al. 2015), holobiont light absorption efficiency (Scheufen et al. 2017a, Krämer et al. 2022), chlorophyll a concentration (Jones 1997), and the rate of respiration, photosynthesis, and calcification (Carlot et al. 2022) can be collected from each sample in real-time. In the same vein, it is important to accurately identify the experimental phenotype, such as degree of bleaching, disease state, life stage, and anything else, i.e. relevant to the study or can confound the results. For instance, recent studies have proposed and confirmed the utility of defining coral bleaching as the phenotype where coral photosynthesis is fully suppressed (Scheufen et al. 2017a,b, Krämer et al. 2022).

However, some crucial information can only be obtained through using colonies maintained and followed for years. This includes a colony’s bleaching history, which has been shown to impact coral and algal metabolism for over 12 months following bleaching (Thomas and Palumbi 2017), its normal adapted conditions (i.e. temperature range, normal pollution levels, and radiation intensity; Grottoli et al. 2020), its seasonal changes (Scheufen et al. 2017a,b), and its reproductive history. For this reason, when possible, it is advised to use colonies with historical data available.

Another essential requirement with coral multiomics studies is that metabolism is quenched in an identical and appropriate manner for every sample. The complete preservation time of coral nubbins varies drastically depending on the method applied (Andersson et al. 2019) and amount of organic versus nonorganic material present, so fragments should be of similar size. Moreover, knowledge concerning the time a chosen metabolic quenching technique takes to thoroughly halt metabolism for the entire sample is useful to consider its effects on the omics techniques employed (i.e. small metabolite turnover rates are faster than RNA, which are faster than proteins and DNA), in addition to which downstream analyses are possible with each preservation technique (McLachlan et al. 2021, Thurber et al. 2022). For example, snap-freezing a small coral fragment in liquid nitrogen will halt metabolism within 10 min, while slow freezing at −20° will take a day, but neither method offer the potential for microscopy or image analyses because the ice crystals disrupt tissue structure (Hagedorn et al. 2013).

Concerning time series experiments, depending on the question asked, the suitable timing between collections is imperative. If a study aims to link mRNA to protein expression levels, the time-scale of the experiment must be minutes to hours, rather than days to weeks (Vogel and Marcotte 2012, Buccitelli and Selbach 2020). Likewise, because corals slough off their mucus as quickly as every 3 hours depending on the species (Bessell-Browne et al. 2017), mucosal microbial diversification studies should also follow a shorter timescale design. In comparison, it has been shown that endosymbiotic algal diversity remains fairly constant (Cunning et al. 2015), so longer time series experiments concerning the symbiont’s response to environmental perturbations are less complicated. Regardless of the experimental design, time points should be chosen with circadian cycles (i.e. daily sample collections should be performed at the same time; Tarzona et al. 2018) and seasonal changes (Scheufen et al. 2017b) in mind.

The necessary experimental conditions to not only illicit a measurable biological response, but a response to realistic stressor intensities, either at current or future levels, should be considered. This will vary depending on each body of water, as well as seasonal changes (Scheufen et al. 2017b) and the exact location of the coral colonies utilized in each experiment (Kelly et al. 2014, Barott et al. 2021). Environmental conditions assayed in coral studies are typically conducted under peak sea surface conditions predicted for the end of the 21st century, whereas models project complex environmental variability (Ziegler et al. 2021). Temperatures will be warmer year-round, with longer warming events during summer months and hourly temperature and *p*CO_2_ fluctuations extending beyond the projected averages corals are expected to face within 100 years (IPCC et al. 2014). For tank experiments, including these diurnal fluctuations would more accurately mimic the holobiont’s response and adaptation to environmental perturbations.

## Conclusion

The coral holobiont possesses broad variation in stress tolerance traits that natural selection can act upon (Wright et al. 2019). Presumably, the microbiome and symbionts encompass a significant portion of such variation in stress tolerance, adaptation, and evolution. The complexity of the holobiont and the ecosystem it creates can benefit from a holistic view captured through multiomics data, but integrating various omics data is not without complications. Experimental constraints, a lack of high-quality genomes, a significant number of dark genes and metabolites, difficulties sequencing and culturing microbial species, analytical challenges, and issues imposed by separating holobiont members are but a few. A major obstacle facing the coral field will be learning how to effectively integrate omics data from all members of the holobiont to understand the system, rather than only its individual parts. However, as our power to generate and analyze multiomics data has grown, it has become clear that there is no one-size fits all approach to understanding holobiont interactions and its resulting health.
